# Home-based traditional Chinese exercise for knee osteoarthritis: a systematic review and meta-analysis of randomized controlled trials

**DOI:** 10.3389/fmed.2025.1665680

**Published:** 2025-09-09

**Authors:** Wenxuan Wang, Haiying Lu, Chuchu Yan, Yawei Shan

**Affiliations:** School of Nursing, Shanghai University of Traditional Chinese Medicine, Shanghai, China

**Keywords:** traditional Chinese exercise, knee osteoarthritis, home-based rehabilitation, systematic review, meta-analysis, randomized controlled trials

## Abstract

**Background:**

Home-based traditional Chinese exercise (TCE) has been proven to be a promising method for improving knee function in patients with knee osteoarthritis (KOA). However, no consensus has been reached among studies on its effectiveness. The study aims to evaluate the effectiveness and safety of the home-based TCE, including Tai Chi, Baduanjin, Yijinjing, and Wuqinxi, for improving knee function, pain, quality of life, and mental health in patients with KOA.

**Methods:**

Eight electronic databases including PubMed, The Cochrane Library, EMBASE, Web of Science, CNKI, Wanfang, VIP, and SinoMed were systematically searched for randomized controlled trials (RCTs) from their inception to February 2025. Two reviewers independently extracted data and assessed methodological quality using the Cochrane Risk of Bias tool. The meta-analysis was conducted using Review Manager 5.4.

**Results:**

Eleven RCTs involving 854 participants were included. TCE showed a significant improvement in general knee function (SMD = −0.61; 95% CI: −1.02 to −0.20; *p* = 0.003), pain (SMD = −0.52; 95% CI: −0.88 to −0.15; *p* = 0.006), the physical quality of life (SMD = −0.41; 95% CI: −0.80 to −0.02; *p* = 0.04), and depression (SMD = −1.00; 95% CI: −1.74 to −0.26; *p* = 0.008) with minimal adverse events. However, data on stiffness and cost-effectiveness were limited.

**Conclusion:**

Home-based TCE is a safe and effective complementary intervention for managing KOA, particularly when integrated into a structured programme combining supervised sessions and home-based practice. Further high-quality RCTs with standardized protocols and longer follow-up are warranted.

**Systematic review registration:**

https://www.crd.york.ac.uk/PROSPERO/view/CRD42024503800, Identifier CRD42024503800.

## 1 Introduction

Knee osteoarthritis (KOA) is the most prevalent chronic and progressive joint disorder, affecting over 650 million individuals globally as of 2020, and ranking as the fourth leading cause of disability worldwide (1). Characterized by joint pain, stiffness, and functional impairment, KOA significantly diminishes quality of life and imposes a considerable burden on both families and healthcare systems (2). Although KOA remains incurable, rehabilitation exercise is widely recognized as the cornerstone of non-pharmacological treatment, with substantial evidence supporting its effectiveness in alleviating pain, improving joint mobility, and slowing disease progression (3, 4).

Among various rehabilitation strategies, home-based exercise plays a pivotal role in KOA management. It enables patients to engage in therapeutic physical activity within their familiar environments, supported by traditional materials (e.g., printed guides, telephone consultations) or digital platforms (e.g., instructional videos, mobile applications) (5). Nevertheless, designing an optimal home-based programme remains challenging due to the need to balance feasibility, accessibility, cost-effectiveness, safety, and patient adherence (6, 7). While routine exercises-such as quadriceps strengthening, straight leg raising, and knee flexion training-have demonstrated efficacy (8), their repetitive nature often leads to diminished long-term adherence. For instance, Pisters et al. reported that only 30.1% of participants maintained exercise routines after 5 years (9).

Traditional Chinese exercise (TCE) offers a promising alternative. Rooted in Traditional Chinese Medicine (TCM), TCE integrates physical, psychological, and social dimensions of health (10). Modalities such as Tai Chi, Baduanjin, Wuqinxi, and Yijinjing have been shown to reduce pain (11), improve sleep quality (12), strengthen musculature (13), and enhance proprioceptive function (14). These practices are generally simple to learn, engaging, low-cost, and require minimal equipment, making them particularly well-suited for elderly KOA patients to perform independently at home (3, 15).

Despite its promise, the effectiveness of home-based TCE for KOA remains unclear. Several systematic reviews have indicated improvements in knee function-including reductions in pain and stiffness-following TCE interventions (16–18). However, findings are inconsistent. For example, Qiu et al. found that Baduanjin did not significantly enhance overall knee function, and that only specific TCE forms (Wuqinxi and Yijinjing) improved stiffness but not pain (10). Such variability may be attributed to heterogeneity in study inclusion and methodology. Similarly, the evidence regarding TCE’s impact on quality of life is inconclusive; while some reviews report no meaningful changes (19), others suggest tangible benefits (10). These discrepancies may arise from differences in the types of TCE evaluated, selection criteria, or search strategies employed (19, 20). Furthermore, prior reviews have largely overlooked TCE’s mindfulness and psychological dimensions, despite its foundation in mind–body integration (16, 21).

In light of these uncertainties, further research is warranted to clarify the efficacy of home-based TCE interventions for KOA. The present systematic review and meta-analysis aims to synthesize current evidence and address existing gaps by evaluating the impact of home-based TCE on knee function and mental health outcomes in KOA patients. Specifically, this study seeks to answer the following questions:

Does home-based TCE significantly improve knee function and well-being in individuals with KOA compared to routine rehabilitation exercises?

What are the essential components of an effective home-based TCE programme for enhancing knee function?

## 2 Materials and methods

This systematic review and meta-analysis was conducted in accordance with the Preferred Reporting Items for Systematic Reviews and Meta-Analyses (PRISMA) 2020 guidelines (22). The study protocol was prospectively registered with the International Prospective Register of Systematic Reviews (PROSPERO), under the registration number CRD42024503800.

### 2.1 Criteria for inclusion and exclusion

#### 2.1.1 Types of studies

The studies of randomized controlled trials (RCTs), cluster RCTs, and interrupted time series studies were included. Excluded were case reports, case series, cross-sectional studies, cohort studies, commentaries, editorials, opinion pieces, reviews, and psychological experiments (e.g., contrastive vignette designs) that do not assess real-life interventions. Duplicate publications and studies for which the full text was entirely inaccessible were also excluded.

#### 2.1.2 Participants

Eligible participants were adults (aged ≥18 years) with a diagnosis of KOA, confirmed via standardized criteria such as the American College of Rheumatology guidelines, radiographic evidence, or physician-confirmed clinical diagnosis. Participants must have been discharged from clinical care and be undergoing home-based rehabilitation. Individuals with a history of knee trauma or surgery, lower limb functional disorders, or severe comorbidities (e.g., cardiopulmonary disease, hepatic or renal dysfunction) were excluded.

#### 2.1.3 Interventions and comparators

Participants were assigned to either an intervention or control group based on the prescribed exercise regimen. Both groups adhered to structured exercise programmes, which included details on objectives, modality, intensity, duration, frequency, total volume, progression, safety considerations, and educational content for home practice. The intervention group engaged in home-based TCE, such as Tai Chi, Baduanjin, Wuqinxi, or Yijinjing. These modalities incorporate coordinated movements, breath control, relaxation, and mindfulness, rooted in TCM principles. Control groups followed routine home-based rehabilitation exercises, typically comprised a combination of aerobic and resistance exercises. Aerobic activities, such as walking or stationary cycling, were generally prescribed at 50–75% of maximum heart rate (HRmax) for 20–40 min per session, performed 3–5 times per week. Resistance training targeted major lower limb muscle groups, using intensities of approximately 50–60% of one-repetition maximum (1RM) or equivalent to a perceived exertion level of 11–13 on the Borg scale for 30–60 min per session, performed 3–4 times per week (23). Progression was achieved by gradually increasing resistance load or session duration.

#### 2.1.4 Outcomes

To be included, studies had to report at least one of the following outcomes: (1) Primary outcomes: Knee function (pain, stiffness, physical function including gait parameters) and safety. These were assessed using validated tools such as the Western Ontario and McMaster Universities Osteoarthritis Index (WOMAC) (24), Visual Analog Scale (VAS) (25), Knee Injury and Osteoarthritis Outcome Score (KOOS) (26), and Hospital for Special Surgery (HSS) knee score (27). Gait was evaluated using the 6 min walk test (28). Safety outcomes included adverse events (e.g., soreness, joint pain, falls, dizziness, dyspnoea, cardiovascular events), or study withdrawals due to health concerns. (2) Secondary outcomes: Exercise adherence, quality of life, psychological well-being, and cost-effectiveness. Adherence was measured via self-report, exercise diaries, telemedicine application logs, behavior or compliance questionnaires, or physiological biofeedback (29). Quality of life and mental health outcomes were evaluated using validated instruments such as the Short-Form 36 (SF-36) (30), Beck Depression Inventory (BDI) (31), and the Center for Epidemiologic Studies Depression Scale (CES-D) (32). Cost-effectiveness was assessed by evaluating participants’ expenditure related to rehabilitation.

### 2.2 Search strategy

A systematic search was performed in eight electronic databases from inception to February 2025: PubMed, The Cochrane Library, EMBASE, Web of Science, and the Chinese databases of CNKI, Wanfang Data, China Science and Technology Journal Database (VIP), and SinoMed. Search strategies included combinations of Medical Subject Headings (MeSH) and free-text terms related to “knee osteoarthritis” and “traditional Chinese exercise,” tailored to the syntax of each database. The complete PubMed search strategy is provided in Supplementary Table 1.

Reference lists of all included studies and relevant systematic reviews were screened manually to identify additional eligible trials. Grey literature, including conference proceedings and trial registries, was searched. Where necessary, study authors were contacted for clarification or additional data.

### 2.3 Study selection and data extraction

All identified records were imported into EndNote X9, where duplicates were automatically removed. Two reviewers (WWX and YCC) independently screened titles and abstracts for relevance, followed by a full-text review based on the inclusion criteria. Discrepancies were resolved by discussion or, when required, consultation with a third reviewer (SYW). Data extraction was independently conducted by two reviewers (WWX and YCC) using a pre-designed extraction form. Extracted data included author name, year of publication, study setting, sample size, participant demographics (e.g., age, sex, group allocation), intervention characteristics (e.g., TCE type, programme content, duration, frequency), follow-up duration, primary and secondary outcomes, and reported adverse events. Disagreements were resolved through discussion.

### 2.4 Risk of bias assessment

Two reviewers (WWX and YCC) independently assessed the risk of bias for each included RCT using the Cochrane Risk of Bias Tool (33). The following seven domains were evaluated: random sequence generation, allocation concealment, blinding of participants and personnel, blinding of outcome assessors, completeness of outcome data, selective outcome reporting, and other sources of bias. Each domain was rated as low, high, or unclear risk of bias. Authors were contacted to obtain missing information, and disagreements were resolved by consensus with a third reviewer (SYW).

### 2.5 Data synthesis and statistical analysis

Where studies reported continuous outcome data, we calculated standardized mean differences (SMDs) with 95% confidence intervals (CIs), to account for variations in scale instruments across trials. Effect sizes were interpreted according to Cohen’s benchmarks (0.2 = small, 0.5 = moderate, 0.8 = large) (34). Meta-analyses were conducted using Review Manager 5.4. The decision to apply fixed- or random-effects models was based on heterogeneity, as assessed by the *χ^2^* test and *I^2^* statistic. A fixed-effects model was applied when *I^2^* < 50% and *p* > 0.10; otherwise, a random-effects model was used (35). In cases of substantial heterogeneity (*I^2^* > 75%) and inconsistent results across forest plots, narrative synthesis was undertaken. Subgroup analyses were conducted based on intervention type (e.g., Tai Chi vs. Baduanjin) and intervention duration to explore potential sources of heterogeneity. Sensitivity analyses were also performed to assess the robustness of the findings. Statistical significance was defined as *p* < 0.05. For analyses including more than five studies, funnel plots were used to visually assess publication bias (36, 37).

## 3 Results

### 3.1 Study selection

**Figure 1:**
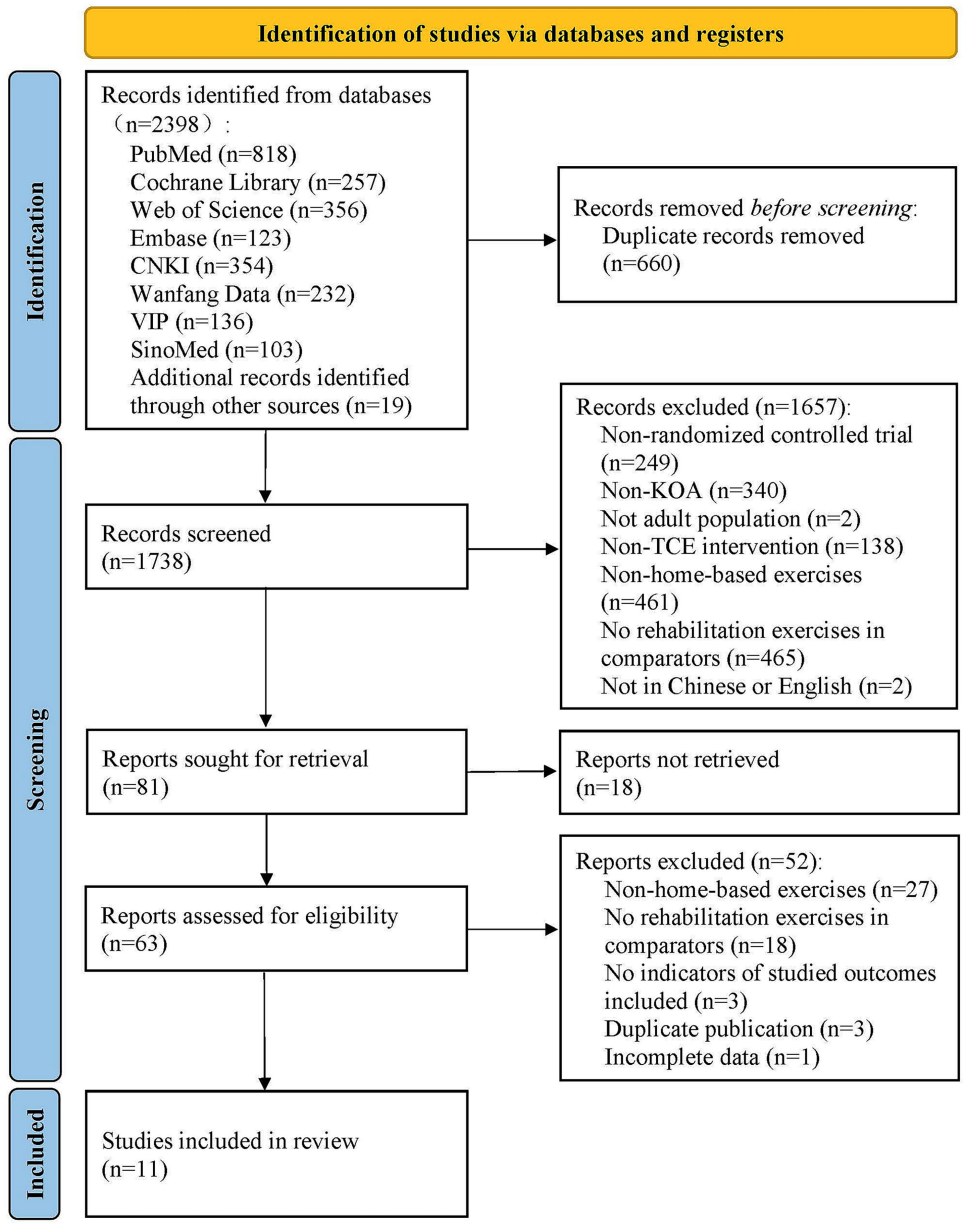
Flowchart of the study selection process.

A total of 2,398 records were initially identified through database searches. After screening titles and abstracts, 81 articles were selected for full-text assessment. Of these, 18 were excluded due to the unavailability of full texts, and 52 were excluded based on eligibility criteria following full-text review. Ultimately, 11 RCTs involving 854 participants were included in the final analysis (38–48). The complete study selection process is illustrated in Figure 1.

### 3.2 Study characteristics

Among the 11 included RCTs, five studies evaluated Tai Chi as the primary intervention (38, 39, 43, 44, 47), one evaluated Baduanjin (46), two focused on Yijinjing (41, 48), and three examined the effects of Wuqinxi (40, 42, 45). The studies were published between 2009 and 2022, and were conducted primarily in China and the United States. The mean age of participants ranged from 42 to 80 years, with a majority of participants being female.

### 3.3 Intervention characteristics

Tai Chi was the most frequently employed form of TCE across the included trials. Interventions typically consisted of instructor-led sessions followed by independent home-based practice. Most studies instructed participants to engage in home exercise for a minimum of 20 min per day, with total weekly durations ranging from 80 to 840 min. The duration of the TCE interventions varied from 3 to 52 weeks, with 12 weeks being the most common. Follow-up periods were generally 12 weeks. Primary outcome domains included pain, stiffness, and physical function. Detailed characteristics of the included studies are presented in Table 1.

**Table 1:** Characteristics of the included studies.

Study (first author/year)	Study location	Participant characteristics				Intervention characteristics					Follow-up (weeks)	Outcomes	Adverse effects
		Grouping	Sample size	Mean age (Mean ± SD, year)	Sex ratio (Female/Male)	Interventions		Time (min/day)	Frequency (times per week)	Duration (weeks)			
Cao (2018) (38)	China	Intervention group	22	70.89 ± 9.80	19/3	Yang-style Tai Chi	Tai Chi sessions + Tai Chi extra exercise^a^ (6 weeks)	60 + 20^a^	1	12	3, 6, 9, 12, 15, 18	①②③④	None
							Tai Chi home exercise (6 weeks)	≥20	7				
		Control group	19	68.89 ± 8.90	15/4	Stretching + wellness education	(Stretching exercise + wellness education) sessions (6 weeks)	20 + 40	2				
							Stretching home exercise (6 weeks)	≥20	7				
Lee (2017) (39)	USA	Intervention group	41	59.90 ± 10.10	NA	Yang-style Tai Chi	Tai Chi sessions (12 weeks)	60	2	12	12, 24	②④⑤⑥⑦⑧	NA
							Tai Chi extra exercise^a^	≥20^a^	7				
		Control group	34	60.90 ± 10.80	NA	Physical therapy	One-to-one physical therapy sessions (6 weeks)	30	2				
							Physical therapy home exercise (6 weeks)	30	4				
							Physical therapy extra exercise^a^	≥20^a^	7				
Li (2021) (40)	China	Intervention group	55	58.51 ± 1.20	37/18	Wuqinxi + isokinetic training + tuina^b^	(Isokinetic training + tuina^b^) in hospital + Wuqinxi home exercise	30 + 30^b^ + 10	4	3	3, 24	②	NA
		Control group	53	57.09 ± 1.22	37/16	Isokinetic training + tuina^b^	(Isokinetic training + tuina^b^) in hospital	30 + 30^b^					
Li (2022) (41)	China	Intervention group	30	63.67 ± 9.39	22/8	Yijinjing	Yijinjing home exercise after wellness education	40	3	12	12	①②③④	None
		Control group	28	62.68 ± 8.87	23/5	Proprioceptive training	Proprioceptive home exercise after wellness education	40	3				
Tang (2019) (42)	China	Intervention group	28	60.36 ± 4.73	19/9	Wuqinxi + isokinetic training + tuina^b^	(Isokinetic training + tuina^b^) in hospital + Wuqinxi home exercise	30 + 30^b^ + 10	4	3	3	②	NA
		Control group	28	59.86 ± 5.92	21/7	Isokinetic training + tuina^b^	(Isokinetic training + tuina^b^) in hospital	30 + 30^b^					
Wang (2009) (43)	USA	Intervention group	20	63.00 ± 8.10	16/4	Yang-style Tai Chi	Tai Chi sessions (12 weeks)	60	2	12	12, 24, 48	②③④⑤⑥⑦⑧	None
							Tai Chi extra exercise^a^	≥20^a^	7				
		Control group	20	68.00 ± 7.00	14/6	Stretching + wellness education	(Stretching exercise + wellness education) sessions (12 weeks)	20 + 40	2				
							Stretching extra exercise^a^	≥20^a^	7				
Wang (2016) (44)	USA	Intervention group	106	60.30 ± 10.50	75/31	Tai Chi	Tai Chi sessions + Tai Chi extra exercise^a^ (12 weeks)	60 + 20^a^	2	52	12, 24, 52	②③④⑤⑥⑦⑧	None
							Tai Chi home exercise (40 weeks)	≥30	7				
		Control group	98	60.10 ± 10.50	68/30	Standard physical therapy	Outpatient physical therapy sessions (6 weeks)	30	2				
							Physical therapy home exercise (6 weeks)	30	4				
							Physical therapy home exercise (40 weeks)	≥30	7				
Xiao (2021) (45)	China	Intervention group	34	70.70 ± 9.36	23/11	Wuqinxi	Wuqinxi home training	60	4	12	12	②③④⑤	NA
		Control group	34	70.20 ± 10.35	22/12	Conventional physical therapy	Resistance + aerobic home training^c^	NA + 30^c^	4				
Yang (2021) (46)	China	Intervention group	50	69.82 ± 4.72	28/27	Horizontal Baduanjin + routine rehabilitation exercises^d^	Routine rehabilitation exercises^d^ + Baduanjin sessions in hospital	60^d^ + 60	14	8	4, 8	①②	NA
							Routine rehabilitation exercises^d^ + Baduanjin exercise at home after discharge						
		Control group	50	71.54 ± 3.12	26/29	Routine rehabilitation exercises^d^	Routine rehabilitation exercises^d^ in hospital	60^d^	14				
							Routine rehabilitation exercises^d^ at home after discharge						
Zeng (2021) (47)	China	Intervention group	31	62.81 ± 8.09	21/10	Yang-style Tai Chi + wellness education	Tai Chi sessions (4 weeks)	60	3	12	4, 8, 12	①②⑤	None
							Tai Chi home exercise (8 weeks)						
							Wellness education on KOA (12 weeks)	60	1/2				
		Control group	30	60.50 ± 6.50	14/16	Quadriceps exercise + wellness education	Quadriceps home exercise (12 weeks)	60	3				
							Wellness education on KOA (12 weeks)	60	1/2				
Zhang (2022) (48)	China	Intervention group	22	55.76 ± 8.37	NA	Yijinjing	Yijinjing sessions (4 weeks)	40	2	12	12	②③④⑥⑦⑧	None
							Yijinjing home exercise (8 weeks)						
		Control group	21	53.40 ± 10.66	NA	Stretching training exercise	Stretching sessions (4 weeks)	40	2				
							Stretching home exercise (8 weeks)						

① The total score of WOMAC; ② WOMAC/VAS pain score; ③ WOMAC stiffness score; ④ WOMAC physical function score; ⑤ the 6 min walk test; ⑥ the physical component summary score of quality of life; ⑦ the mental component summary score of quality of life; ⑧ BDI/CES-D depression score.

a Participants were additionally asked to practice relevant exercise at home for at least 20 min per day.

b Tuina is an ancient Chinese manual therapy that integrates various techniques, including pressing, kneading, rolling, and stretching, to loosen soft tissues like muscles and ligaments, reduce joint load, and improve blood circulation.

c The resistance training was performed using three sets per exercise at intensities between 6 and 12 RM (repetition maximum). The aerobic training was conducted at 75–85% of heart rate.

d Routine rehabilitation exercises included ankle pump, quadriceps isometric contraction, straight leg raising, lower limb flexion-extension, and chest expansion exercises, etc.

BDI, The Beck Depression Inventory; CES-D, The Center for Epidemiologic Studies Depression Scale; NA, not reported; VAS, The Visual Analog Scale; WOMAC, The Western Ontario and McMaster Universities Osteoarthritis Index.

### 3.4 Risk of bias assessment

Risk of bias was assessed and summarized in Supplementary Figures 1, 2. Adequate random sequence generation was reported in nine studies (38–40, 42, 43, 45–48). Allocation concealment was adequately described in five studies (39, 44, 45, 47, 48). Only one study was rated as low risk for performance bias, with explicit blinding of participants and personnel (48). High risk of detection bias was noted in two studies due to involvement of outcome assessors in the intervention process without reporting blinding procedures (46, 47). Attrition bias was judged as unclear in two studies due to incomplete reporting of outcome data (41, 47). One study was found to have high reporting bias due to selective outcome reporting (39). Eight studies were rated as unclear for other potential sources of bias due to insufficient information (40–46, 48).

### 3.5 Meta-analysis of primary outcomes

**Figure 2:**
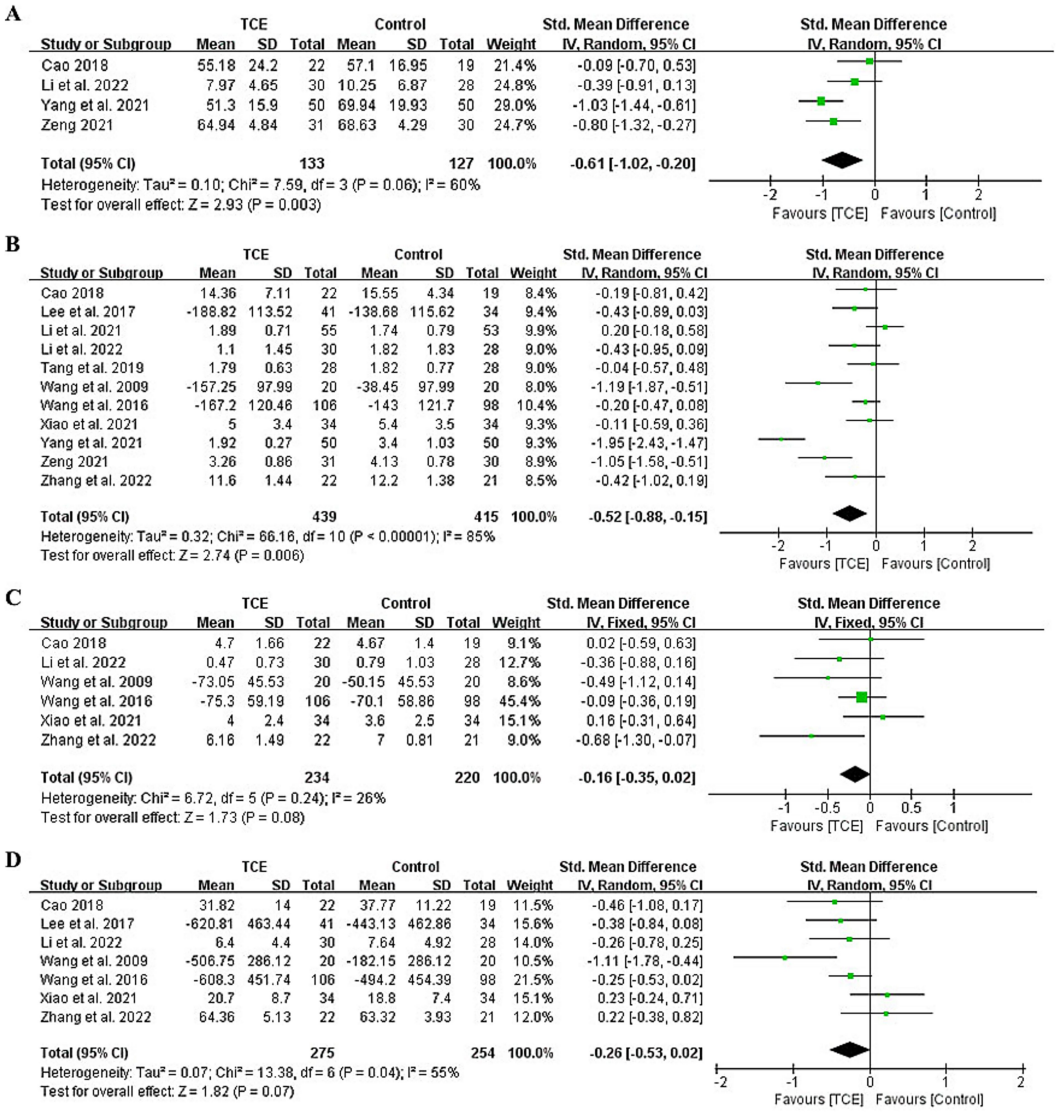
Forest plot of effects on WOMAC. **(A)** Forest plot of effects on the total score of WOMAC. **(B)** Forest plot of effects on the pain score of WOMAC/VAS. **(C)** Forest plot of effects on the stiffness score of WOMAC. **(D)** Forest plot of effects on the physical function score of WOMAC. VAS, The Visual Analog Scale; WOMAC, The Western Ontario and McMaster Universities Osteoarthritis Index.

Four studies comprising 260 participants assessed the effects of TCE versus routine rehabilitation on the general knee function with total WOMAC score (38, 41, 46, 47). A random-effects model was applied due to substantial heterogeneity (*I^2^* = 60%, *p* = 0.06). TCE interventions were associated with significantly greater improvements in general knee function with a moderate effect size (SMD = −0.61; 95% CI: −1.02 to −0.20; *p* = 0.003) (Figure 2A).

All 11 included studies (*n* = 854) evaluated pain using the WOMAC or VAS (38–48). The pooled results demonstrated that TCE significantly reduced pain compared to control interventions with a moderate effect size (SMD = −0.52; 95% CI: −0.88 to −0.15; *p* = 0.006) (Figure 2B). However, high heterogeneity was observed (*I^2^* = 85%). Subgroup analyses were conducted to explore sources of heterogeneity. When stratified by TCE type, the ranking of effectiveness for pain reduction was Baduanjin > Tai Chi > Yijinjing > Wuqinxi, with significant differences favoring Baduanjin (SMD = −1.95; 95% CI: −2.43 to −1.47; *p* < 0.001), Tai Chi (SMD = −0.57; 95% CI: −0.96 to −0.18; *p* = 0.005), and Yijinjing (SMD = −0.43; 95% CI: −0.82 to −0.03; *p* = 0.03). The statistically significant subgroup differences (*χ^2^_3_* = 52.36, *P* for interaction < 0.001) strongly supported this finding (Supplementary Figure 3A).

Duration-based subgroup analysis categorized studies into interventions lasting <12 weeks and ≥12 weeks. Interventions of ≥12 weeks demonstrated significantly better outcomes for pain relief (SMD = −0.46; 95% CI: −0.71 to −0.21; *p* = 0.0004). However, the test for subgroup differences was not statistically significant (*χ^2^_1_* = 0.04, *P* for interaction = 0.84), indicating that the observed effect size differences might not represent a true modifying effect of intervention duration (Supplementary Figure 3B).

Six studies involving 454 participants reported WOMAC stiffness outcomes (38, 41, 43–45, 48). A fixed-effects model was used due to low heterogeneity (*I^2^* = 26%, *p* = 0.24). The meta-analysis found no statistically significant difference between TCE and control groups with a small effect size (SMD = −0.16; 95% CI: −0.35 to 0.02; *p* = 0.08), however, the direction of effect favored TCE (Figure 2C).

Seven studies with 529 participants evaluated physical function using the WOMAC scale (38, 39, 41, 43–45, 48). A random-effects model revealed no statistically significant difference with a small effect size (SMD = −0.26; 95% CI: −0.53 to 0.02; *p* = 0.07), with moderate heterogeneity (*I^2^* = 55%, *p* = 0.04), however, the direction of effect favored TCE (Figure 2D). Five studies involving 448 participants reported 6 min walk test outcomes (39, 43–45, 47). The meta-analysis indicated no statistically significant improvement associated with TCE with a large effect size (SMD = −0.80; 95% CI: −1.64 to 0.05; *p* = 0.06), while the direction of effect favored TCE (Supplementary Figure 4).

### 3.6 Meta-analysis of secondary outcomes

**Figure 3:**
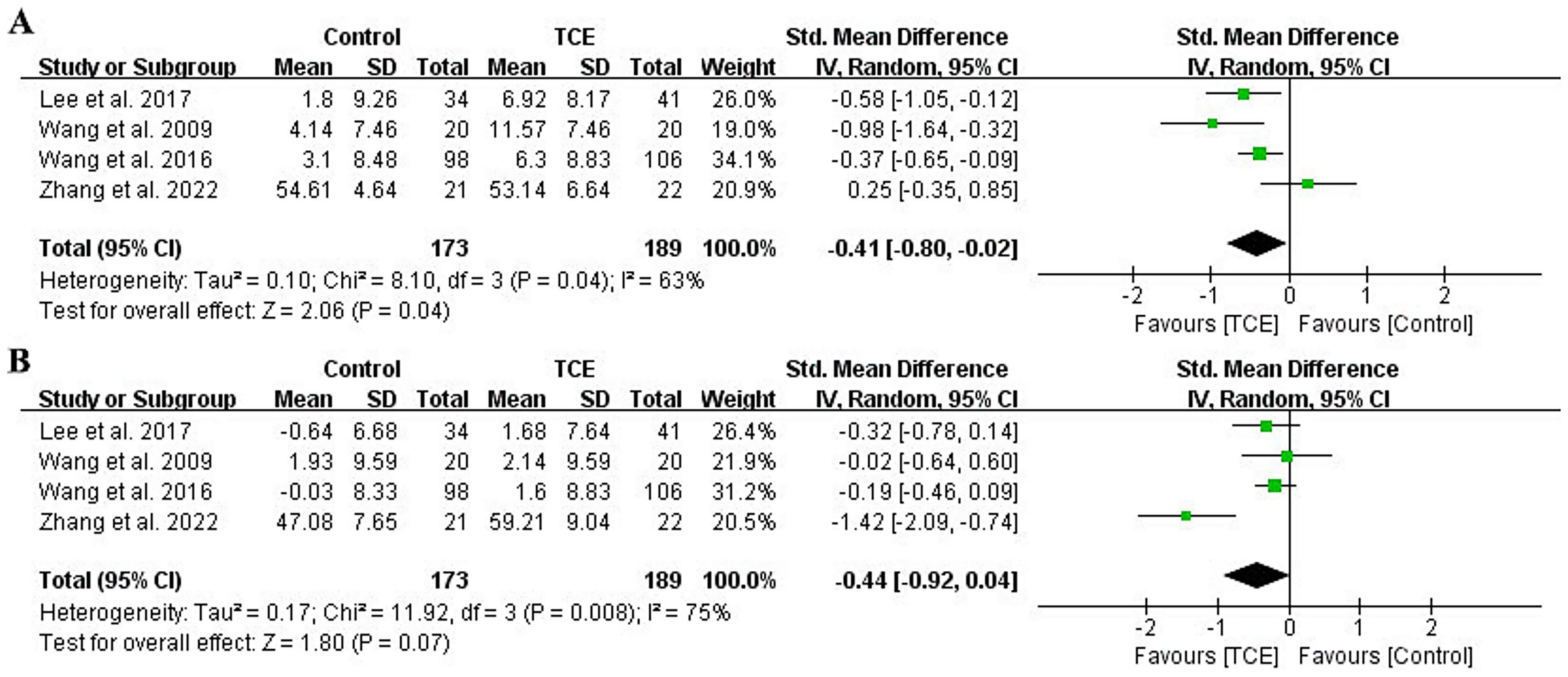
Forest plot of effects on the quality of life (SF-36). **(A)** Forest plot of effects on the physical component summary score of quality of life (SF-36). **(B)** Forest plot of effects on the mental component summary score of quality of life (SF-36). SF-36, The Short-Form 36.

Four studies (*n* = 362) assessed quality of life using the SF-36 or equivalent scales (39, 43, 44, 48). A random-effects model indicated that TCE significantly improved physical quality of life yielding a small effect size (SMD = −0.41; 95% CI: −0.80 to −0.02; *p* = 0.04), with moderate heterogeneity (*I^2^* = 63%) (Figure 3A). No statistically significant effect was observed in mental quality of life with a small effect size (SMD = −0.44; 95% CI: −0.92 to 0.04; *p* = 0.07) with substantial heterogeneity (*I^2^* = 75%), however, the direction of effect favored TCE (Figure 3B).

**Figure 4:**
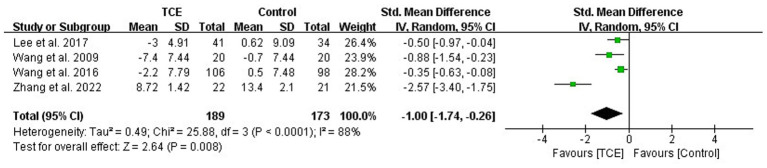
Forest plot of effects on the depression score of BDI/CES-D. BDI, The Beck Depression Inventory; CES-D, The Center for Epidemiologic Studies Depression Scale.

Four studies (*n* = 362) examined depressive symptoms (39, 43, 44, 48). TCE interventions were associated with significantly greater reductions in depression scores with a large effect size (SMD = −1.00; 95% CI: −1.74 to −0.26; *p* = 0.008), although high heterogeneity was observed (*I^2^* = 88%) (Figure 4). Subgroup analyses by TCE type revealed that both Tai Chi (SMD = −0.46; 95% CI: −0.71 to −0.22; *p* = 0.0002) and Yijinjing (SMD = −2.57; 95% CI: −3.40 to −1.75; *p* < 0.001) were more effective than control interventions in reducing depressive symptoms. The statistically significant subgroup differences (*χ^2^_1_* = 23.06, *P* for interaction <0.001) strengthened this conclusion (Supplementary Figure 5).

Adverse events were monitored via participant self-report. No serious adverse events related to TCE were reported. Minor adverse effects, such as transient muscle soreness and joint discomfort, were reported in four studies approximately 2–3% of participants during the early stages of intervention (38, 43, 47, 48).

### 3.7 Sensitivity analysis

Sensitivity analysis on general knee function was conducted by excluding the study with the greatest weight (46), which resulted in reduced heterogeneity (*I^2^* = 35%) without altering the overall effect size, suggesting robustness of the findings (Supplementary Table 2). After excluding the largest study (44) from the physical quality of life analysis, heterogeneity increased (*I^2^* = 75%) and the effect was no longer statistically significant, indicating that the result may be sensitive to the influence of individual studies (Supplementary Table 3). This study had the largest sample size (*n* = 106), a 52-week Tai Chi intervention duration, and used the SF-36 questionnaire to assess quality of life. Its extended intervention duration, high adherence rates, and use of a sensitive outcome measurement tool likely amplified observed effects, thereby exerting a disproportionate influence on the pooled estimate. This study was also excluded from the depression analysis, and did not substantially affect the results on mental health (*I^2^* = 89%), confirming the stability of the observed effect (Supplementary Table 4).

### 3.8 Publication bias

Funnel plot asymmetry was noted for outcomes with ≥5 studies, suggesting the presence of potential publication bias (Supplementary Figure 6).

## 4 Discussion

This systematic review and meta-analysis identified a growing body of literature, published primarily between 2016 and 2022, focused on the application of TCE for KOA, with most studies originating from China and the United States. This trend reflects the influence of national health policies and international collaborations. For instance, the Healthy China 2030 initiative, launched in 2016, advocated for the integration of physical activity and medical care (49), while the American College of Rheumatology officially recommended Tai Chi as a non-pharmacological therapy for KOA (3). Moreover, generalisability to other regions may be influenced by cultural familiarity with TCE, availability of trained instructors, and integration of TCE into healthcare systems. In countries where TCE is less culturally embedded, patient uptake may be lower, and adaptations may be required to fit local preferences and infrastructure (49). Despite the growing attention on TCE, the methodological quality of included RCTs requires improvement, particularly in aspects such as allocation concealment, blinding procedures, and sample size calculation. Moreover, behavioral interventions like TCE are susceptible to cross-contamination, which may compromise the internal validity of the results.

This review demonstrated that TCE significantly improved knee function and alleviated pain, with minimal adverse effects. Tai Chi in particular was effective in reducing joint inflammation and improving lower limb muscle strength, especially in the quadriceps. These physiological improvements can enhance joint stability, reduce biomechanical load, and subsequently decrease pain and improve daily functional capacity (8, 50). A 12-week Tai Chi regimen has also been shown to lower serum inflammatory markers, aligning with the intervention duration used in many of the included studies (8, 11, 47). However, heterogeneity in TCE styles, such as Yang, Sun, and Wu styles, and variations in form (e.g., 8-form, 12-form, 24-form), presents challenges for standardizing protocols (37). In addition, heterogeneity may stem from other components of exercise protocols such as movement complexity, intensity, meditative focus, and targeted muscle groups. For example, Tai Chi involves continuous, weight-bearing movements that may enhance quadriceps strength, whereas Baduanjin and Yijinjing may place greater emphasis on flexibility and mindfulness. Some control groups received active rehabilitation (40, 42), while others received minimal care, potentially influencing effect sizes (46). Future research should explore the specific mechanisms and optimal movement sequences for KOA rehabilitation (47). Furthermore, adequate instruction and preparatory sessions are essential to ensure correct technique and therapeutic effect. Group-based instruction further enhances accuracy and adherence. To support home-based practice, participants were provided with instructional videos and materials, passed a performance assessment, and were followed up regularly via phone or *WeChat* (a multi-faceted mobile messaging app) to monitor compliance and adverse events.

Subgroup analyses suggested that both Baduanjin and Yijinjing were effective in reducing pain, though results varied due to the limited number of studies (10, 21). Horizontal Baduanjin, performed in a supine position, may be especially suitable for patients with limited mobility (46). Conversely, Wuqinxi did not demonstrate statistically significant effects on pain reduction. This finding should be interpreted with caution due to the limited number of studies (*n* = 3) assessing this intervention. Furthermore, Wuqinxi protocols varied in duration and intervention periods, and in some cases, participants in the control groups received concurrent tuina therapy, which could have reduced between-group differences (40, 42). The small sample sizes and combined therapies may have diluted potential effects, limiting the strength of conclusions.

Furthermore, no consistent improvements were observed in stiffness or physical function, which contrasts with previous studies (16, 21). Such discrepancies may be attributed to variability in control interventions; this review only included studies comparing TCE with routine rehabilitation. Furthermore, although some studies used the WOMAC index to assess knee function, others reported only the total score or omitted it while providing subscale scores for pain, stiffness, and physical function (39, 40, 42–45, 48). This led to smaller sample sizes in the studies, reducing statistical power. However, the absence of significant effects for stiffness and physical function may also reflect a true lack of benefit or insensitivity of measurement tools, although improvements in pain and mobility contributed to enhanced physical quality of life, echoing findings by Qiu et al. (10). Longer intervention durations or more sensitive outcome measures may be required to detect changes in these domains. Additionally, TCE interventions were linked to reduced depressive symptoms, likely due to the synergistic effects of aerobic exercise, meditative focus, and pain alleviation (15). However, no studies provided comprehensive data on cost-effectiveness, highlighting a gap in the literature. Future studies should incorporate economic evaluations to determine the cost-effectiveness of TCE, particularly in comparison to standard rehabilitation or pharmacological management.

From a clinical perspective, moderate improvements in knee function and pain reduction, combined with large improvements in depressive symptoms, suggest that TCE may offer meaningful benefits for patients with KOA, especially when integrated into home-based rehabilitation programmes. Based on current evidence, an effective TCE intervention for KOA should incorporate the following components: (1) Pre-home training phase: At least 4 weeks of professionally guided TCE sessions (2–3 times/week, 40–60 min/session), including movement assessments to ensure accuracy. (2) Sustained home exercise phase: A minimum of 6 weeks of home practice, with at least 20 min per session and a total of ≥150 min per week. Comprehensive instructional materials should be provided. (3) Minimum duration: A total intervention period of 12 weeks is recommended to optimize functional outcomes. (4) Ongoing monitoring: Regular follow-ups via eHealth tools or outpatient visits are advised to support adherence, monitor progress, and evaluate long-term effectiveness.

Despite the strengths of this review-including its comprehensive inclusion of recent literature in both English and Chinese and detailed analysis of intervention formats-several limitations remain. High heterogeneity among studies may introduce bias. The small number of studies investigating Baduanjin, Yijinjing, and Wuqinxi limits the reliability of subgroup findings. Variation in follow-up periods, with only initial post-intervention data analyzed, precluded long-term outcome assessments.

## 5 Conclusion

This systematic review suggests that home-based TCE, particularly Tai Chi, Baduanjin, and Yijinjing, are effective, safe, and feasible non-pharmacological interventions for managing KOA. TCE significantly improved knee function and pain relief, with minimal adverse events, especially when applied over a 12-week period with structured guidance and sustained home practice. Some studies also reported improvements in physical quality of life and depressive symptoms. However, variations in TCE types, intervention protocols, and methodological quality of the included trials limit the generalisability of findings. The current evidence base is weakened by moderate-to-low study quality and limited data on stiffness, physical function, and cost-effectiveness. Future research should focus on high-quality RCTs with standardized protocols, longer follow-up, and economic evaluations. Overall, TCE shows promise as a complementary therapy for KOA and may be integrated into clinical and community rehabilitation programmes to support personalized, culturally relevant care.

## Data Availability

The datasets presented in this study can be found in online repositories. The names of the repository/repositories and accession number(s) can be found in the article/Supplementary material.

## References

[1] Cui A Li H Wang D Zhong J Chen Y Lu H. Global, regional prevalence, incidence and risk factors of knee osteoarthritis in population-based studies. EClinicalMedicine. (2020) 29–30:100587. doi: 10.1016/j.eclinm.2020.100587 PMC7704420 34505846

[2] Collaborators GO. Global, regional, and national burden of osteoarthritis, 1990–2020 and projections to 2050: a systematic analysis for the global burden of disease study 2021. Lancet Rheumatol. (2023) 5:e508–22. doi: 10.1016/s2665-9913(23)00163-7 37675071 PMC10477960

[3] Kolasinski SL Neogi T Hochberg MC Oatis C Guyatt G Block J . 2019 American College of Rheumatology/Arthritis Foundation guideline for the Management Of Osteoarthritis Of The Hand, Hip, And Knee. Arthritis Rheumatol. (2020) 72:220–33. doi: 10.1002/art.41142 31908163 PMC10518852

[4] Brophy RH Fillingham YA. AAOS clinical practice guideline summary: Management of Osteoarthritis of The Knee (Nonarthroplasty), Third Edition. J Am Acad Orthop Surg. (2022) 30:e721–9. doi: 10.5435/jaaos-d-21-01233 35383651

[5] Si J Sun L Li Z Zhu W Yin W Peng L. Effectiveness of home-based exercise interventions on pain, physical function and quality of life in individuals with knee osteoarthritis: a systematic review and meta-analysis. J Orthop Surg Res. (2023) 18:503. doi: 10.1186/s13018-023-04004-z 37461112 PMC10351144

[6] van Doormaal MCM Meerhoff GA Vliet Vlieland TPM Peter WF. A clinical practice guideline for physical therapy in patients with hip or knee osteoarthritis. Musculoskeletal Care. (2020) 18:575–95. doi: 10.1002/msc.1492 32643252

[7] Rausch Osthoff AK Niedermann K Braun J Adams J Brodin N Dagfinrud H . 2018 EULAR recommendations for physical activity in people with inflammatory arthritis and osteoarthritis. Ann Rheum Dis. (2018) 77:1251–60. doi: 10.1136/annrheumdis-2018-213585 29997112

[8] Peng J. (2021) Effect of Taichi training on functional rehabilitation of knee joint after total knee arthroplasty. [Master's thesis]. China: Guangzhou University of Chinese Medicine.

[9] Pisters MF Veenhof C Schellevis FG Twisk JW Dekker J De Bakker DH. Exercise adherence improving long-term patient outcome in patients with osteoarthritis of the hip and/or knee. Arthritis Care Res (Hoboken). (2010) 62:1087–94. doi: 10.1002/acr.20182 20235201

[10] Qiu B Wang W Tang G Chai S Zhang X Zhou P . Long- and short-term effectiveness of traditional Chinese exercises in improving the overall physical capacity of patients with knee osteoarthritis: a systematic review and meta-analysis. Medicine (Baltimore). (2024) 103:e39520. doi: 10.1097/md.0000000000039520 39252253 PMC11383713

[11] Liu J Chen L Chen X Hu K Tu Y Lin M . Modulatory effects of different exercise modalities on the functional connectivity of the periaqueductal grey and ventral tegmental area in patients with knee osteoarthritis: a randomised multimodal magnetic resonance imaging study. Br J Anaesth. (2019) 123:506–18. doi: 10.1016/j.bja.2019.06.017 31395306

[12] Lü J Huang L Wu X Fu W Liu Y. Effect of Tai Ji Quan training on self-reported sleep quality in elderly Chinese women with knee osteoarthritis: a randomized controlled trail. Sleep Med. (2017) 33:70–5. doi: 10.1016/j.sleep.2016.12.024 28449910

[13] Chen PY Song CY Yen HY Lin PC Chen SR Lu LH . Impacts of tai chi exercise on functional fitness in community-dwelling older adults with mild degenerative knee osteoarthritis: a randomized controlled clinical trial. BMC Geriatr. (2021) 21:449. doi: 10.1186/s12877-021-02390-9 34332537 PMC8325845

[14] Hu X Lai Z Wang L. Effects of Taichi exercise on knee and ankle proprioception among individuals with knee osteoarthritis. Res Sports Med. (2020) 28:268–78. doi: 10.1080/15438627.2019.1663520 31524502

[15] Xu Z. (2023) The effect of standard Tai Chi Chuan on exercise ability and happiness index in overweight adults with exercise deficiency: an exploratory research. [Master's thesis]. China: China Academy of Chinese Medical Sciences

[16] Zhang S Huang R Guo G Kong L Li J Zhu Q . Efficacy of traditional Chinese exercise for the treatment of pain and disability on knee osteoarthritis patients: a systematic review and meta-analysis of randomized controlled trials. Front Public Health. (2023) 11:1168167. doi: 10.3389/fpubh.2023.1168167 37361162 PMC10285305

[17] Zeng ZP Liu YB Fang J Liu Y Luo J Yang M. Effects of Baduanjin exercise for knee osteoarthritis: a systematic review and meta-analysis. Complement Ther Med. (2020) 48:102279. doi: 10.1016/j.ctim.2019.102279 31987253

[18] Guo J Peng C Hu Z Guo L Dai R Li Y. Effect of Wu Qin Xi exercises on pain and function in people with knee osteoarthritis: a systematic review and meta-analysis. Front Med (Lausanne). (2022) 9:979207. doi: 10.3389/fmed.2022.979207 36419784 PMC9676488

[19] Zhang Y Huang L Su Y Zhan Z Li Y Lai X. The effects of traditional Chinese exercise in treating knee osteoarthritis: a systematic review and Meta-Analysis. PLoS One. (2017) 12:e0170237. doi: 10.1371/journal.pone.0170237 28121996 PMC5266306

[20] Tan B Yan Y Zhou Q Ran Q Chen H Sun S . Kinesitherapy for knee osteoarthritis patients physical and psychological health based on "traditional Chinese exercise" management modalities: a systematic review and Meta-Analysis of randomized controlled trials. Orthop Surg. (2024) 16:3–16. doi: 10.1111/os.13920 38018392 PMC10782256

[21] Li R Chen H Feng J Xiao Y Zhang H Lam CW . Effectiveness of traditional Chinese exercise for symptoms of knee osteoarthritis: a systematic review and Meta-Analysis of randomized controlled trials. Int J Environ Res Public Health. (2020) 17:7873. doi: 10.3390/ijerph17217873 33121082 PMC7662219

[22] Page MJ McKenzie JE Bossuyt PM Boutron I Hoffmann TC Mulrow CD . The PRISMA 2020 statement: an updated guideline for reporting systematic reviews. BMJ. (2021) 372:n71. doi: 10.1136/bmj.n71 33782057 PMC8005924

[23] Wilson RC Jones PW. Long-term reproducibility of Borg scale estimates of breathlessness during exercise. Clin Sci (Lond). (1991) 80:309–12. doi: 10.1042/cs0800309 1851065

[24] Bellamy N Buchanan WW Goldsmith CH Campbell J Stitt LW. Validation study of WOMAC: a health status instrument for measuring clinically important patient relevant outcomes to antirheumatic drug therapy in patients with osteoarthritis of the hip or knee. J Rheumatol. (1988) 15:1833–40. 3068365

[25] Hjermstad MJ Fayers PM Haugen DF Caraceni A Hanks GW Loge JH . Studies comparing numerical rating scales, verbal rating scales, and visual analogue scales for assessment of pain intensity in adults: a systematic literature review. J Pain Symptom Manag. (2011) 41:1073–93. doi: 10.1016/j.jpainsymman.2010.08.016 21621130

[26] Roos EM Roos HP Lohmander LS Ekdahl C Beynnon BD. Knee Injury and Osteoarthritis Outcome Score (KOOS)--development of a self-administered outcome measure. J Orthop Sports Phys Ther. (1998) 28:88–96. doi: 10.2519/jospt.1998.28.2.88 9699158

[27] Słupik A Białoszewski D. Comparative analysis of clinical usefulness of the Staffelstein Score and the Hospital for Special Surgery Knee Score (HSS) for evaluation of early results of total knee arthroplasties. Preliminary report. Ortop Traumatol Rehabil. (2007) 9:627–35. 18227754

[28] King S Wessel J Bhambhani Y Maikala R Sholter D Maksymowych W. Validity and reliability of the 6 minute walk in persons with fibromyalgia. J Rheumatol. (1999) 26:2233–7. 10529146

[29] Zhao Y Tang F Yang A Wang B. Some methods of measuring patients adherence to medication. Chin Pharm J. (2013) 48:1308–10.

[30] Brazier JE Harper R Jones NM O'Cathain A Thomas KJ Usherwood T . Validating the SF-36 health survey questionnaire: new outcome measure for primary care. BMJ. (1992) 305:160–4. doi: 10.1136/bmj.305.6846.160 1285753 PMC1883187

[31] Beck AT Steer RA Ball R Ranieri W. Comparison of Beck depression inventories -IA and -II in psychiatric outpatients. J Pers Assess. (1996) 67:588–97. doi: 10.1207/s15327752jpa6703_13 8991972

[32] Lewinsohn PM Seeley JR Roberts RE Allen NB. Center for Epidemiologic Studies Depression Scale (CES-D) as a screening instrument for depression among community-residing older adults. Psychol Aging. (1997) 12:277–87. doi: 10.1037//0882-7974.12.2.277 9189988

[33] Nasser M. Cochrane handbook for systematic reviews of interventions. Am J Public Health. (2020) 110:753–4. doi: 10.2105/ajph.2020.305609

[34] Parker RI Hagan-Burke S. Useful effect size interpretations for single case research. Behav Ther. (2007) 38:95–105. doi: 10.1016/j.beth.2006.05.002 17292698

[35] Riley RD Higgins JP Deeks JJ. Interpretation of random effects meta-analyses. BMJ. (2011) 342:d549. doi: 10.1136/bmj.d549 21310794

[36] Peters JL Sutton AJ Jones DR Abrams KR Rushton L. Comparison of two methods to detect publication bias in meta-analysis. JAMA. (2006) 295:676–80. doi: 10.1001/jama.295.6.676 16467236

[37] Zeng L Yang W Guo D Luo M Pan J Han Y . System evaluation of traditional exercise therapy intervention on pain and joint function improvement in patients with knee osteoarthritis. China J Tradit Chin Med Pharm. (2018) 33:2132–9.

[38] Cao X. (2018) The effects of Tai Chi practice in elderly subjects with knee osteoarthritis. [Master's thesis]. China: Guangxi University of Chinese Medicine

[39] Lee AC Harvey WF Wong JB Price LL Han X Chung M . Effects of Tai Chi versus physical therapy on mindfulness in knee osteoarthritis. Mindfulness (N Y). (2017) 8:1195–205. doi: 10.1007/s12671-017-0692-3 28959369 PMC5612617

[40] Li C Tang L Lin B Zhang K Li Z Zhou G . Effect of isokinetic training combined with Wu Qin-xi on muscle strength of knee osteoarthritis and long-term effect. Clin J Tradit Chin Med. (2021) 33:157–61. doi: 10.16448/j.cjtcm.2021.0138

[41] Li Y Ye Y Niu X Qiu Z Zhong W. Effects of Yi Jin Jing exercise on the coordination activation ability of lower limb muscles of knee osteoarthritis. China J Tradit Chin Med Pharm. (2022) 37:2380–5.

[42] Tang L Li C Zhang K Xiao A Pan Z Lin Y. Clinical efficacy observation of massage combined with isokinetic training and Wuqinxi on knee osteoarthritis. J Hunan Univ Chin Med. (2019) 39:879–84.

[43] Wang C Schmid CH Hibberd PL Kalish R Roubenoff R Rones R . Tai Chi is effective in treating knee osteoarthritis: a randomized controlled trial. Arthritis Rheum. (2009) 61:1545–53. doi: 10.1002/art.24832 19877092 PMC3023169

[44] Wang C Schmid CH Iversen MD Harvey WF Fielding RA Driban JB . Comparative effectiveness of Tai Chi versus physical therapy for knee osteoarthritis: a randomized trial. Ann Intern Med. (2016) 165:77–86. doi: 10.7326/m15-2143 27183035 PMC4960454

[45] Xiao CM Li JJ Kang Y Zhuang YC. Follow-up of a Wuqinxi exercise at home programme to reduce pain and improve function for knee osteoarthritis in older people: a randomised controlled trial. Age Ageing. (2021) 50:570–5. doi: 10.1093/ageing/afaa179 32931545

[46] Yang C Gao X Zhan Y Yuan Y Wang Y. Effect of new horizontal Baduanjin characteristic training on elderly patients with knee osteoarthritis. Tradit Chin Med Rehabil. (2021) 12:27–9. doi: 10.19787/j.issn.1008-1879.2021.23.008

[47] Zeng F. (2021) The clinical outcome of Yang’s Tai Chi exercise on knee osteoarthritis. [Master’s thesis]. China: Guangxi University of Chinese Medicine

[48] Zhang S Guo G Li X Yao F Wu Z Zhu Q . The effectiveness of traditional Chinese Yijinjing qigong exercise for the patients with knee osteoarthritis on the pain, dysfunction, and mood disorder: a pilot randomized controlled trial. Front Med (Lausanne). (2022) 8:792436. doi: 10.3389/fmed.2021.792436 35087846 PMC8787110

[49] Chen S. Optimization strategy of physical and medical integration based on "Healthy China 2030" initiative. Phys Educ Rev. (2023) 42:42–4.

[50] Mauri C Cerulli C Grazioli E Minganti C Tranchita E Scotto di Palumbo A . Role of exercise on pain, functional capacity, and inflammatory biomarkers in osteoarthritis: a systematic review and meta-analysis. Ann Phys Rehabil Med. (2025) 68:101909. doi: 10.1016/j.rehab.2024.101909 39798216

