# Supplementary material for: Home-based traditional Chinese exercise for knee osteoarthritis: a systematic review and meta-analysis of randomized controlled trials
Source: Front Med (Lausanne). 2025 Sep 9;12:1665680. doi: 10.3389/fmed.2025.1665680 (PMC12454440; doi:10.3389/fmed.2025.1665680)
Supplement: Supplementary file 2 [file Supplementary_file_1.docx]

***Supplementary Material***

**1 Supplementary Figures**


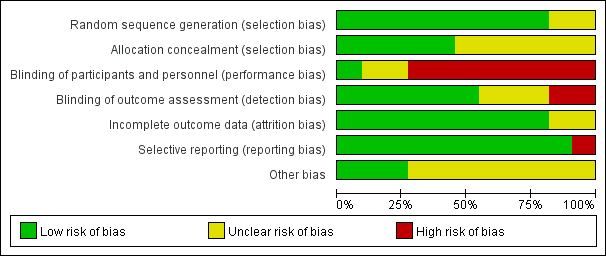


**Supplementary Figure 1.** Risk of bias graph.


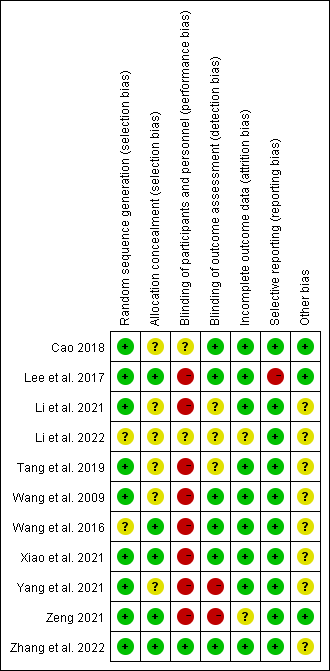


**Supplementary Figure 2.** Risk of bias summary.


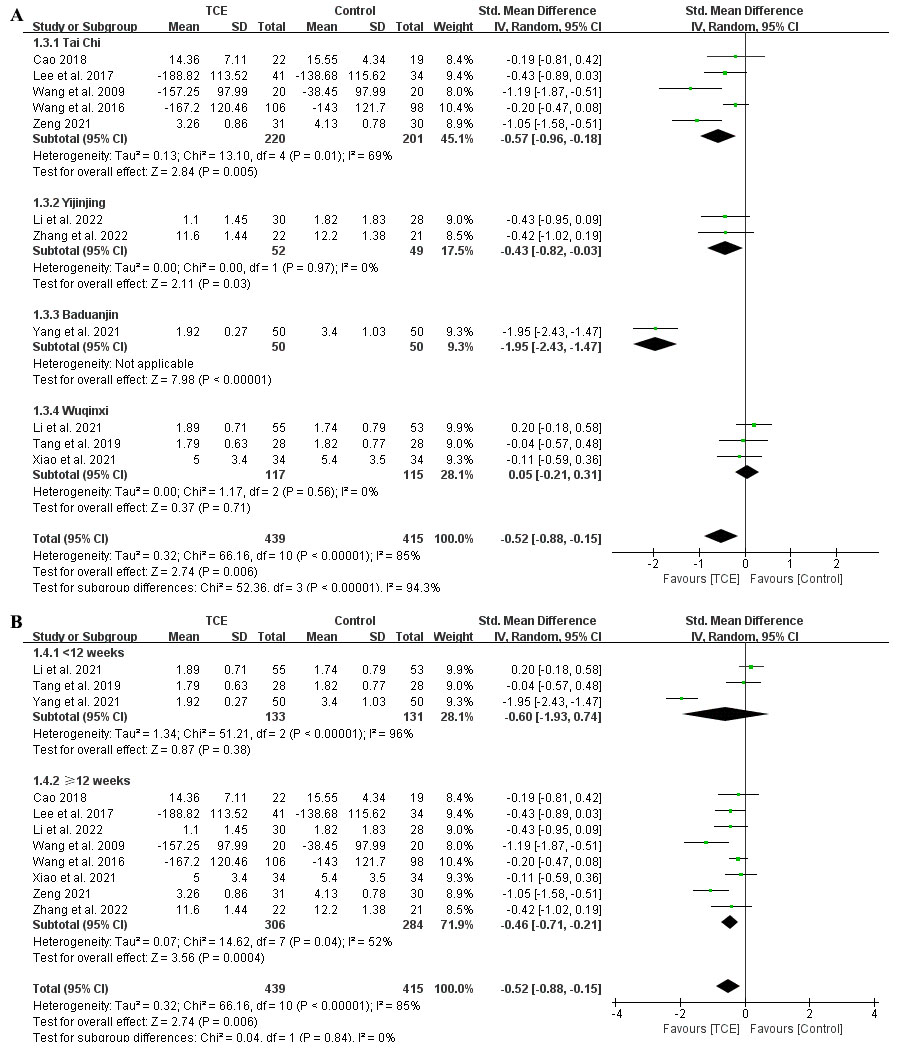


**Supplementary Figure 3.** Forest plot of subgroup analyses on the pain score.

1. Forest plot of subgroup analyses of different intervention types on the pain score. **(B)** Forest plot of subgroup analyses of different intervention duration on the pain score.

**
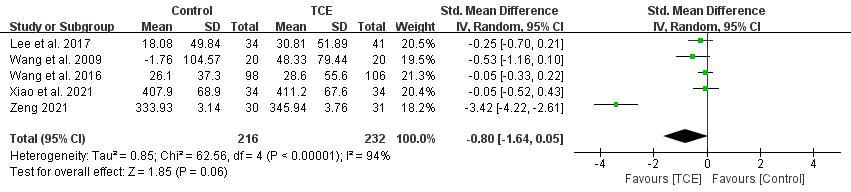
**

**Supplementary Figure 4.** Forest plot of effects on the 6-minute walk test.

**
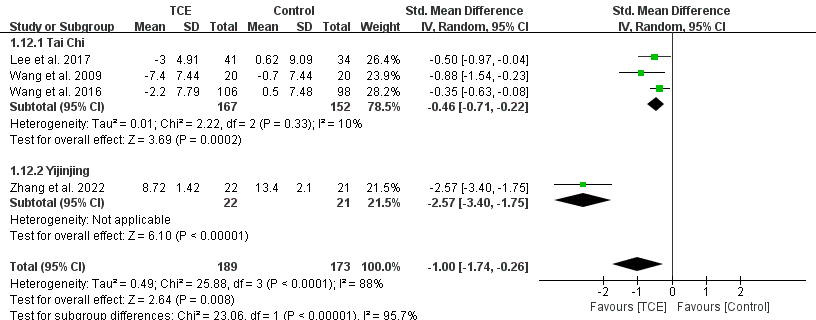
**

**Supplementary Figure 5.** Forest plot of subgroup analyses of different intervention types on mental health.


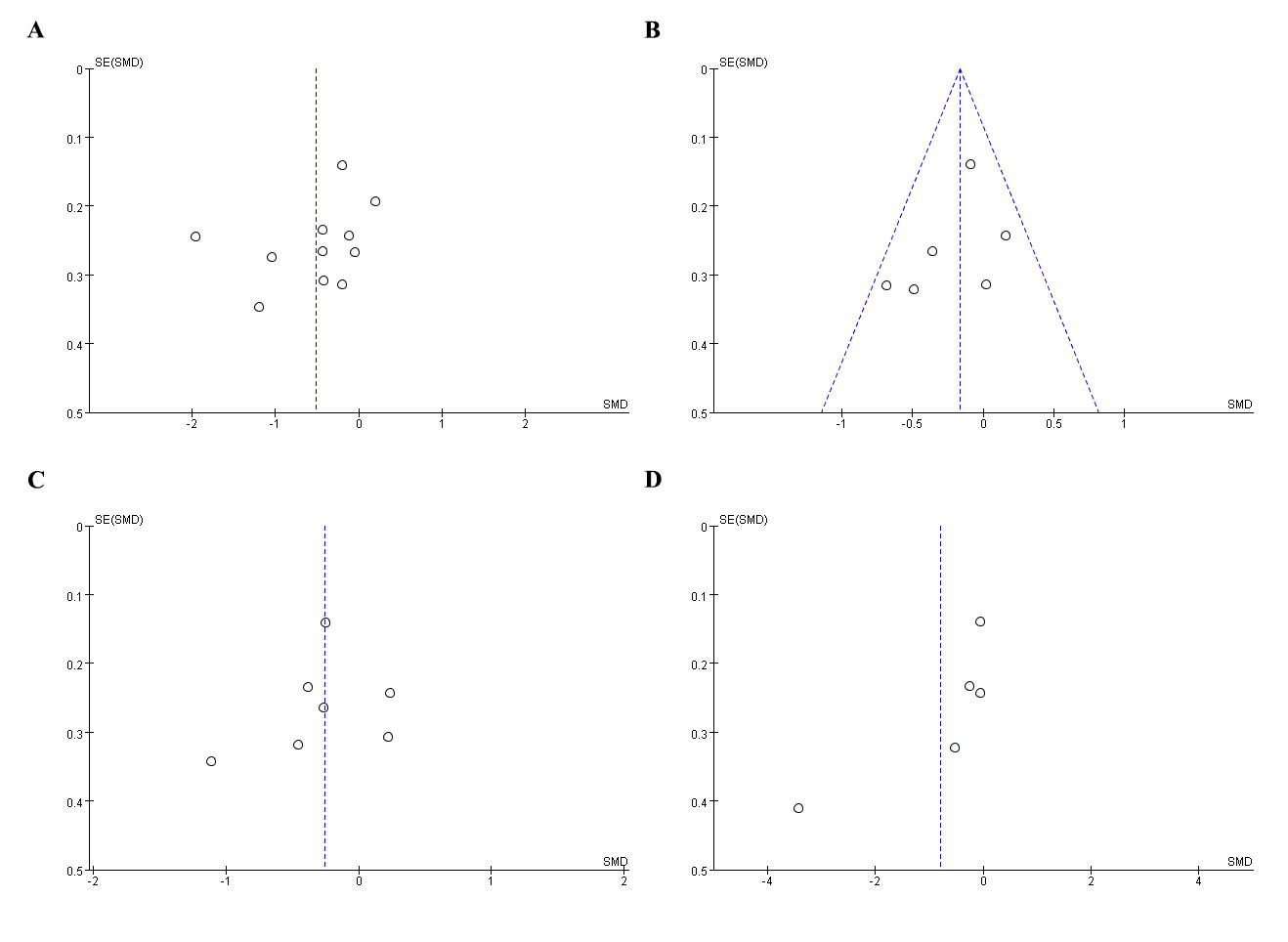


**Supplementary Figure 6.** Funnel plots on the outcomes of this review.

**(A)** Funnel plot of WOMAC/VAS pain score. **(B)** Funnel plot of WOMAC stiffness score. **(C)** Funnel plot of WOMAC physical function score. **(D)** Funnel plot of 6-minute walk test.

VAS, The Visual Analog Scale; WOMAC, The Western Ontario and McMaster Universities Osteoarthritis Index.

**2 Supplementary Tables**

**Supplementary Table 1.** Strategy for searching RCTs in PubMed.

| **Steps** | **Search strategy** |
| --- | --- |
| #1 | "Osteoarthritis, Knee"[MeSH] |
| #2 | "Osteoarthritis, Knee"[Title/Abstract] OR "Knee Osteoarthritides"[Title/Abstract] OR "Knee Osteoarthritis"[Title/Abstract] OR "Osteoarthritis of Knee"[Title/Abstract] OR "Osteoarthritis of the Knee"[Title/Abstract] |
| #3 | #1 OR #2 |
| #4 | "Tai Ji"[Mesh] OR "Qigong"[MeSH] |
| #5 | "Tai Ji"[Title/Abstract] OR "Qigong"[Title/Abstract] OR "Tai Chi"[Title/Abstract] OR "Ch'i Kung"[Title/Abstract] OR "Baduanjin"[Title/Abstract] OR "Eight trigrams boxing"[Title/Abstract] OR "Eight-sectioned exercise"[Title/Abstract] OR "Wuqinxi"[Title/Abstract] OR "Five-animal boxing"[Title/Abstract] OR "Five-animal exercises"[Title/Abstract] OR "Yijinjing"[Title/Abstract] OR "Tendon Change Classic"[Title/Abstract] OR "Liuzijue"[Title/Abstract] OR "Six-character formula"[Title/Abstract] OR "Traditional Chinese exercise"[Title/Abstract] OR "Traditional exercise therapy"[Title/Abstract] |
| #6 | #4 OR #5 |
| #7 | #3 AND #6 |

**Supplementary Table 2.** Comparison of sensitivity analysis of the WOMAC total score.

| Outcome | Sensitivity analysis | Heterogeneity | SMD (95%CI) | *P*-value |
| --- | --- | --- | --- | --- |
| WOMAC total score | Before | *I2*=60%, *P*=0.06 | -0.61[-1.02, -0.20] | *P*=0.003** |
|  | After | *I^2^*=35%, *P*=0.22 | -0.45[-0.84, -0.05] | *P*=0.03* |

**P*<0.05, ***P*<0.01;

WOMAC, The Western Ontario and McMaster Universities Osteoarthritis Index.

**Supplementary Table 3.** Comparison of sensitivity analysis of the PCS score of quality of life (SF-36).

| Outcome | Sensitivity analysis | Heterogeneity | SMD (95%CI) | *P*-value |
| --- | --- | --- | --- | --- |
| SF-36 (PCS) | Before | *I^2^*=63%, *P*=0.04 | -0.41[-0.80, -0.02] | *P*=0.04* |
|  | After | *I^2^*=75%, *P*=0.02 | -0.43[-1.09, 0.23] | *P*=0.20 |

**P*<0.05, ***P*<0.01;

PCS, physical component summary; SF-36, The Short-Form 36.

**Supplementary Table 4.** Comparison of sensitivity analysis of the depression score (BDI/CES-D).

| Outcome | Sensitivity analysis | Heterogeneity | SMD (95%CI) | *P*-value |
| --- | --- | --- | --- | --- |
| BDI/CES-D | Before | *I^2^*=88%, *P* < 0.0001 | -1.00[-1.74, -0.26] | *P*=0.008** |
|  | After | *I^2^*=89%, *P* < 0.0001 | -1.28[-2.39, -0.16] | *P*=0.02* |

**P*<0.05, ***P*<0.01;

BDI, The Beck Depression Inventory; CES-D, The Center for Epidemiologic Studies Depression Scale.
